# Mosquito metallomics reveal copper and iron as critical factors for *Plasmodium* infection

**DOI:** 10.1371/journal.pntd.0009509

**Published:** 2021-06-23

**Authors:** Krystal Maya-Maldonado, Victor Cardoso-Jaime, Gabriela González-Olvera, Beatriz Osorio, Benito Recio-Tótoro, Pablo Manrique-Saide, Iram Pablo Rodríguez-Sánchez, Humberto Lanz-Mendoza, Fanis Missirlis, Fidel de la Cruz Hernández-Hernández

**Affiliations:** 1 Departamento de Infectómica y Patogénesis Molecular, Cinvestav, Ciudad de México, México; 2 Centro de Investigaciones sobre Enfermedades Infecciosas, Instituto Nacional de Salud Pública, Cuernavaca, Morelos, México; 3 Unidad Colaborativa para Bioensayos Entomológicos, Campus de Ciencias Biológicas y Agropecuarias, Universidad Autónoma de Yucatán, Mérida, Yucatán, México; 4 Departamento de Fisiología, Biofísica y Neurociencias, Cinvestav, Ciudad de México, México; 5 Instituto de Biotecnología, Universidad Nacional Autónoma de México, Cuernavaca, Morelos, México; 6 Laboratorio de Fisiología Molecular y Estructural, Facultad de Ciencias Biológicas, Universidad Autónoma de Nuevo León, Monterrey, Nuevo León, México; Kenya Agricultural and Livestock Research Organization, KENYA

## Abstract

Iron and copper chelation restricts *Plasmodium* growth *in vitro* and in mammalian hosts. The parasite alters metal homeostasis in red blood cells to its favor, for example metabolizing hemoglobin to hemozoin. Metal interactions with the mosquito have not, however, been studied. Here, we describe the metallomes of *Anopheles albimanus* and *Aedes aegypti* throughout their life cycle and following a blood meal. Consistent with previous reports, we found evidence of maternal iron deposition in embryos of *Ae*. *aegypti*, but less so in *An*. *albimanus*. Sodium, potassium, iron, and copper are present at higher concentrations during larval developmental stages. Two *An*. *albimanus* phenotypes that differ in their susceptibility to *Plasmodium berghei* infection were studied. The susceptible *white stripe* (*ws*) phenotype was named after a dorsal white stripe apparent during larval stages 3, 4, and pupae. During larval stage 3, *ws* larvae accumulate more iron and copper than the resistant *brown stripe* (*bs*) phenotype counterparts. A similar increase in copper and iron accumulation was also observed in the susceptible *ws*, but not in the resistant *bs* phenotype following *P*. *berghei* infection. Feeding *ws* mosquitoes with extracellular iron and copper chelators before and after receiving *Plasmodium*-infected blood protected from infection and simultaneously affected follicular development in the case of iron chelation. Unexpectedly, the application of the iron chelator to the *bs* strain reverted resistance to infection. Besides a drop in iron, iron-chelated *bs* mosquitoes experienced a concomitant loss of copper. Thus, the effect of metal chelation on *P*. *berghei* infectivity was strain-specific.

## Author summary

To establish a life cycle between insect and mammalian hosts, the malaria parasite has evolved mechanisms to manage metal ions from the distinct microenvironments it encounters. Previous work has addressed how interference using metal chelation affects parasite development in human, primate, and rodent hosts. Similar studies in mosquito species that harbor *Plasmodium* have not been performed. Here, we address such micronutrient relationships in three steps. First, we characterized how the metallome fluctuates during development in two species of mosquito. Second, we asked whether susceptibility to *Plasmodium* infection correlated with a differential response in mosquito metal homeostasis. Third, we tested the effects of iron and copper chelation treatment of adult mosquitoes concerning propensity of infection and mosquito reproduction. Metal ions offer a promising target in the ongoing efforts to control the mosquito-borne disease.

## Introduction

The role of mosquitoes as vectors of different diseases has long been recognized [1]. Many strategies have been applied for vector control [2], with recent additions including genetic strategies [3,4], manipulation of the *Wolbachia* endosymbiont [5], and proposals to influence mosquito behavior [6]. A thorough characterization of the physiological interactions between viruses or parasites and their insect hosts could further inform the design of interventions to interrupt infections in affected areas [7,8]. One such relatively unexplored area of insect physiology–with the possible exception of studies in the model organism *Drosophila melanogaster* [9–13]–is metal metabolism. Several studies exist for iron metabolism in mosquitoes (reviewed in [14]), including characterizations of the iron storage ferritin complex [15], the iron trafficking transferrin protein [16], and iron regulatory proteins [17] in *Aedes aegypti*. Αn iron regulatory protein has also been characterized in *Culex pipiens* [18]. A divalent metal transporter has been identified in *Anopheles albimanus* [19], but appears to be lacking in *Ae*. *aegypti*, where instead homologues of the Zip/ZnT families of transporters may have taken over cellular iron import [20,21]. Heme oxygenase was described in *Anopheles gambiae* [22] and *Ae*. *aegypti* [23].

In *Ae*. *aegypti*, the fate of blood meal iron was traced showing iron accumulation in female ovaries [24,25]. It is also worth noting that *Wolbachia* interferes with insect iron metabolism [26,27]. The kynurenine pathway metabolite, xanthurenic acid, has been proposed to function as an iron chelator [28]. It is also a critical inducer of *Plasmodium* gametogenesis in the intestinal lumen of *Anopheles stephensi* mosquitoes [29]. Amongst the many unknowns in insect iron metabolism [30], recent efforts to identify a heme transporter [31] and unravel the function of multicopper oxidases [32,33] in iron trafficking have been unsuccessful (for related studies in *D*. *melanogaster* see [34–36]). Beyond iron, there has been little attention to other essential metals, such as copper, zinc, manganese, and molybdenum in mosquito biology. Metallothionein gene expression has been studied in *Culex quinquefasciatus* [37], *An*. *gambiae* [38] and *Ae*. *aegypti* [39], whereas several studies have used copper at concentrations where the metal becomes toxic to *Ae*. *aegypti* and *Aedes albopictus* mosquitoes [40–44]. Lower concentrations of copper– 0.15 ppm, 0.30 ppm, and 0.60 ppm for *Ae*. *albopictus*, *An*. *stephensi*, and *C*. *pipiens*, respectively–resulted in developmental delays [45]. Considering that the studies mentioned above have not measured metal content in the mosquitoes, we have determined the metallomes of *Ae*. *aegypti* and *An*. *albimanus* throughout the different stages of their life cycle.

We opted to work with two laboratory-maintained *Ae*. *aegypti* strains, one originally collected in the Carribean in the 1930s known as the Rockefeller (Rock) strain [46,47] and another originally collected in New Orleans in the 1980s known as the New Orleans (NO) strain [48]. The Rock strain is susceptible to dengue virus infection [49]. Recently, host serum iron was shown to modulate dengue virus acquisition [50]. Differences between susceptible and refractory strains have been attributed to a host of immune-related genes. Transferrin is upregulated in the refractory strain [49] and following *in vitro* arboviral infection [51], consistent with a function of this circulating high-affinity iron binding protein [52,53] playing a role in the defense against bacterial infections [16,54,55]. We also chose to work with two *An*. *albimanus* phenotypes, both isolated through inbreeding from a parental strain collected on human bait in the coastal plains of Southern México [56]. The two phenotypes differ with respect to a stripe of white pigment prominent during larval stages 3–4 and pupae, a variable character first noted for this species in 1967 [57] and thought to be composed of uric acid crystals [58]. The phenotypes also differ in their susceptibility to both *P*. *vivax* [56] and *P*. *berghei* [59]. We refer to the susceptible phenotype as *white stripe* (*ws*) due to the stripe’s presence, whereas the refractory (resistant) phenotype has been called *brown stripe* (*bs*). Field studies suggest that *An*. *albimanus* is only second to *Anopheles pseudopunctipennis* in *Anopheles* species population density in México, the two species together accounting for 92% of all individuals sampled [60].

In malaria, the role for iron has long been studied and discussed, although the emphasis has been placed on the parasite and its interaction with the human host (for reviews see [61,62]). The iron chelator desferrioxamine was shown to restrict malaria growth *in vitro* [63,64], in rodent [65,66], and non-human primate [67] hosts. The utility of iron chelators for human treatment is more controversial [61,62,68,69]. A recent population genetics association study suggested that *Plasmodium* may itself cause iron deficiency to the human host [70]. On the other hand, Rasoloson *et al*. studied *P*. *falciparum* in human erythrocytes, assessing copper’s role during the trophozoite stage of the parasite [71]. These authors described that infected erythrocytes had 6.6 ± 2.4 μM copper compared to 10.0 ± 2.3 μM in their uninfected controls. Application of 150 μM bathophenanthroline sulphate (BCS), an extracellular copper chelator, had no effect to copper accumulation in the infected erythrocytes nor to parasite growth. In contrast, 10 μM neocuprine, an intracellular copper chelator, applied to synchronized cultures of early rings, blocked the ring-to-trophozoite transition. More recently, Kenthirapalan *et al*. demonstrated that neocuprine blocked sexual differentiation of *P*. *berghei* and, hence, transmission to the mosquito vector [72]. Study of a *P*. *berghei* mutant for a copper P-type ATPase transporter offered additional evidence in support of the conclusion that parasite copper homeostasis is critical to the parasite in the mosquito vector and to a smaller extent in a rodent host [73]. Thus, an appreciation exists that *Plasmodium* protists require iron and copper to grow and differentiate; however, metal interactions have not been studied in the mosquito host. Here, we have assessed how the *An*. *albimanus* metallome is affected by *P*. *berghei*, using the susceptible *ws* and refractory *bs* phenotypes of the *An*. *albimanus* Tapachula strain [59], and applied extracellular iron and copper chelators to the mosquitoes’ diet, before and after an infected blood meal, to test the impact of metal chelation on the ability of *P*. *berghei* for intestinal colonization.

## Results

### Phenotypic description of the *An*. *albimanus* mosquito strains

**Fig 1:**
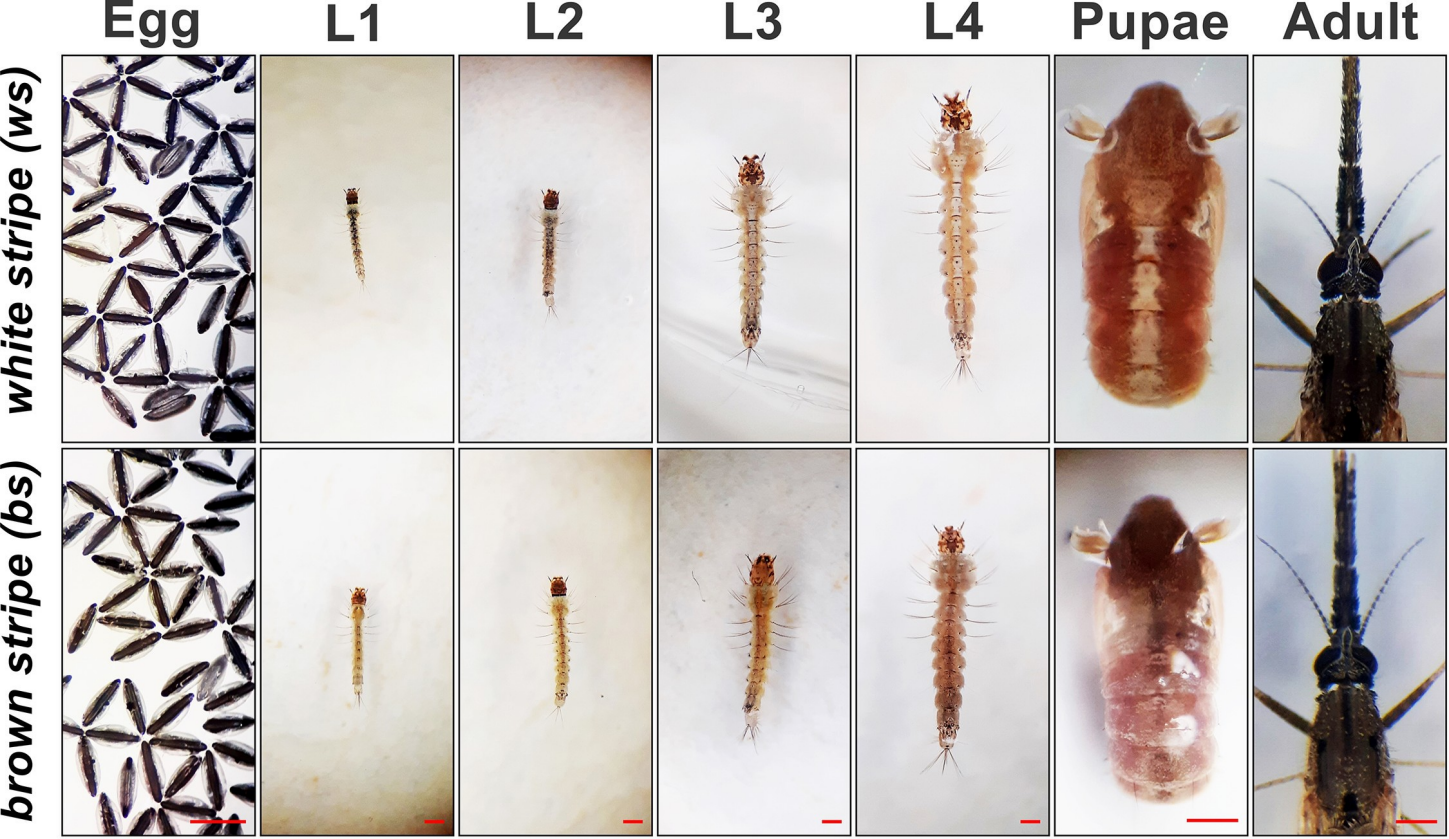
*An*. *albimanus* ontogeny of *ws* and *bs* striped phenotypes. The phenotypic differences (white or brown stripe along the dorsal thorax and abdomen) are only visible from the 3^rd^ instar larval stage until the pupa stage. Adult photographs were obtained from female mosquitoes three days post-emergence. Pupae are from the first 12 hours. Scale bar = 500 μm.

The mosquito life cycle is divided into different stages of development (Fig 1). Adult females lay their eggs on water, where embryonic development proceeds without nutrient acquisition. Four larval stages, during which the insect feeds and grows, are all aquatic. Metamorphosis takes place at the pupa stage. Adult mosquitoes live and reproduce on the land and in the air. Females require a blood meal from a warm-blooded animal for oogenesis.

The *An*. *albimanus* mosquitoes used in this study originate from the Tapachula strain, which shows phenotypic variability to dorsal cuticle pigmentation (S1 Fig). The *ws* and *bs* phenotypes have been isolated previously through successive generations of inbreeding, resulting in stable colonies. Adult mosquitoes, eggs, and the first two larval stages are visually indistinguishable between *ws* and *bs*. In contrast, a prominent white stripe is readily recognizable starting at larval stage 3 and present during stage 4 and in pupae of *ws* mosquitoes; this stripe is brown in the *bs* phenotype (Fig 1).

### Metallomics of *Ae*. *aegypti* and *An*. *albimanus* throughout their life cycle

**Fig 2:**
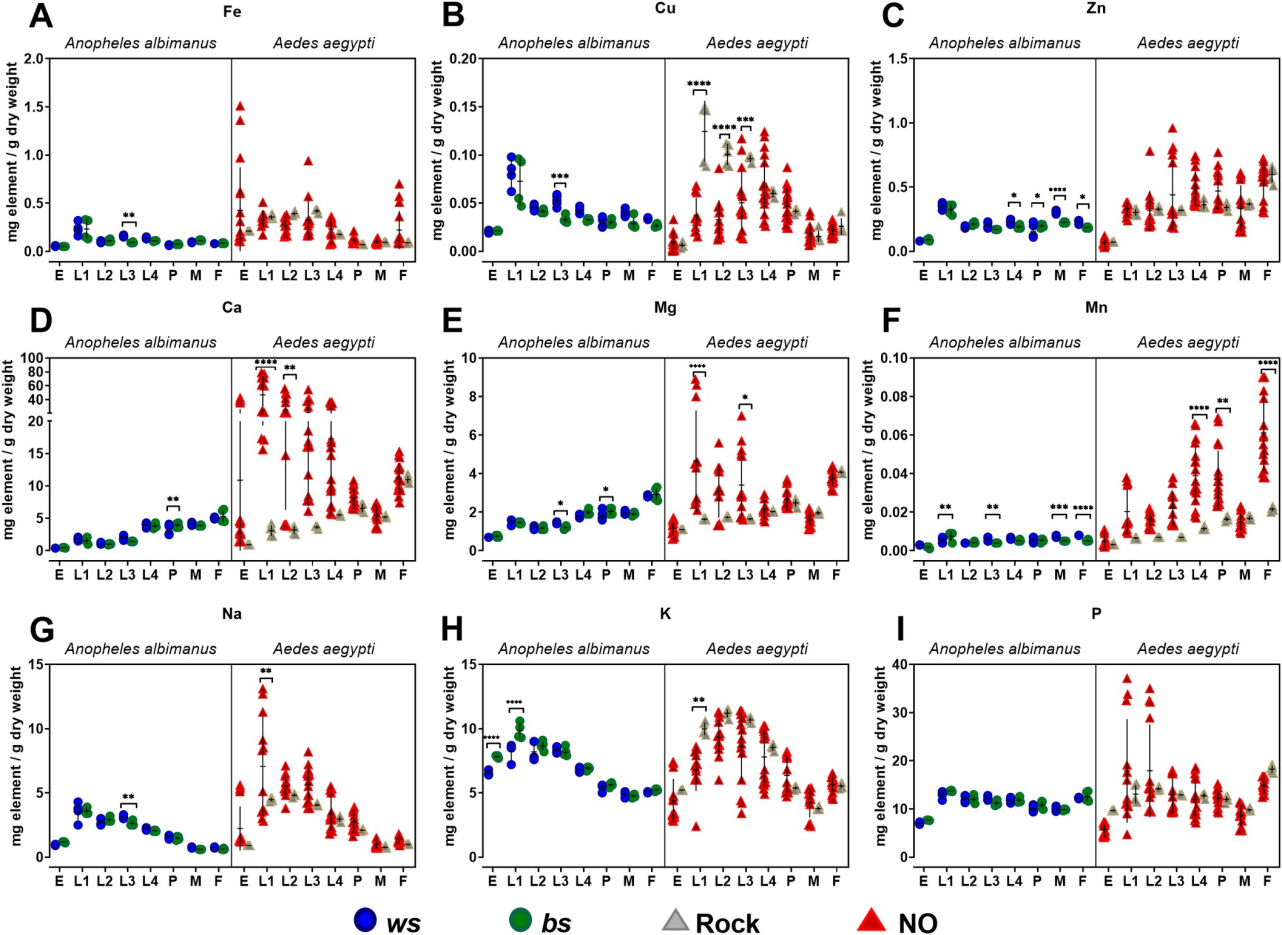
Metallomics during the life cycle of *An*. *albimanus* and *Ae*. *aegypti*. Metal concentrations were determined by ICP-OES in *ws* (blue circles) and *bs* (green circles) phenotypes from *An*. *albimanus*, Rock (grey triangles) and NO (red triangles) strains from *Ae*. *aegypti*, and throughout their life cycle. Panels show different metal ions measured simultaneously in the same samples. A) Iron. B) Copper. C) Zinc. D) Calcium. E) Magnesium. F) Manganese. G) Sodium. H) Potassium. I) Phosphorus. Letters on the X-axis represent instar/stage as follows: E = eggs, L1-L4 = instar 1–4 larvae, P = Pupae, M = Adult male, F = Adult female. Data are expressed as mean ± standard deviation (SD) from several independent measurements taken from two to four biological replicates. Data were tested for normality by the Shapiro-Wilk test and statistical analisys was performed by two-way ANOVA followed by *post-hoc* Bonferroni-corrected test comparing the *ws* to *bs* phenotypes and the Rock to NO strains at each stage. For further comparisons see S1 Table.

We determined the metallomes (nine elements: iron, copper, zinc, calcium, magnesium, manganese, sodium, potassium, and phosphorus, in whole-body samples) in the *ws* and *bs* phenotypes from *An*. *albimanus* and in the Rock and NO strains from *Ae*. *aegypti* throughout their life cycle (Fig 2). The data allow multiple comparisons: between metals, between species, between strains/phenotypes of the same species, between developmental stages within a given species or strain, and for any given metal. We provide the raw data and several statistical analyses supporting statements made below (S1 Table).

Larval stages for both species of mosquitoes concentrate significantly more sodium, potassium, copper, and iron than adult stages. For *Ae*. *aegypti*, the average increase (mean value of all larval stages compared to mean value of adult males and females combined) was five-fold for sodium; three-fold for copper, and two-fold for iron and potassium; in this species, calcium also showed a two-fold increase. For *An*. *albimanus*, the average increase was four-fold for sodium and 1.5-fold for iron, potassium, and copper. Little difference was observed between larvae and adult mosquitoes, adapted to different environments and physiologies, for the rest of the elements (Fig 2).

A notable difference in the metallomes of the two species was observed in maternal-to-egg iron provision. *Ae*. *aegypti* embryos show, as expected [24,25], substantive accumulation of iron, averaging at 0.38 mg per g dry weight, however *An*. *albimanus* embryos only accumulated 0.05 mg per g dry weight (Fig 2A). Notably, copper is deposited in a reverse manner, at 0.008 mg per g dry weight in *Ae*. *aegypti* embryos *versus* 0.021 mg per g dry weight in *An*. *albimanus* embryos (Fig 2B). Otherwise, the developmental patterns observed in the metallomes are well conserved between the two species (Fig 2).

With regards to differences within each species, we observed that in *An*. *albimanus* third instar larvae, a stage during which the *ws* phenotype first appears (Fig 1), larvae of the *ws* phenotype accumulate 72% more iron, 63% more manganese, 61% more copper, and 19% more sodium and magnesium, compared to their *bs* phenotype counterparts (Fig 2). In the adult stages, manganese remained 52% higher, and zinc now showed a moderate 28% increase. Calcium increased throughout the life cycle in both colonies from *An*. *albimanus* and in the *Ae*. *aegypti* Rock strain, whereas, in contrast, it accumulated dramatically during the larval stages in the NO strain (Fig 2D). This change in the developmental pattern of calcium accumulation was one of the differences observed between the Rock and NO strains, another being a three-fold increase in manganese accumulation in the NO strain (compared to Rock) observed during the larval stages and in adult female mosquitoes (Fig 2F). Female NO mosquitoes accumulated four times more manganese than males, but the sex-specific difference was particular to the NO strain. Conserved sex-specific differences (observed in all populations studied here) included almost two-fold increases of calcium and magnesium in females *versus* males (Fig 2D and 2E). A third difference between the Rock and NO strains was observed specifically during the first larval instar stage where the NO strain accumulates calcium, magnesium, and sodium. In contrast, the Rock strain accumulates copper and potassium, instead (Fig 2). Some of these differences between the Rock and NO strains could be attributable to having been raised on different diets (S2 Table). For example increased magnesium and manganese correlate with higher presence of these metals in the diet, whereas increased calcium in the NO strain was despite larvae feeding on a diet with lower calcium concentration. Collectively, the results suggest a fair degree of conservation between species and strains, bar the already mentioned exceptions.

### *P*. *berghei* infection affects the *An*. *albimanus* metallome

**Fig 3:**
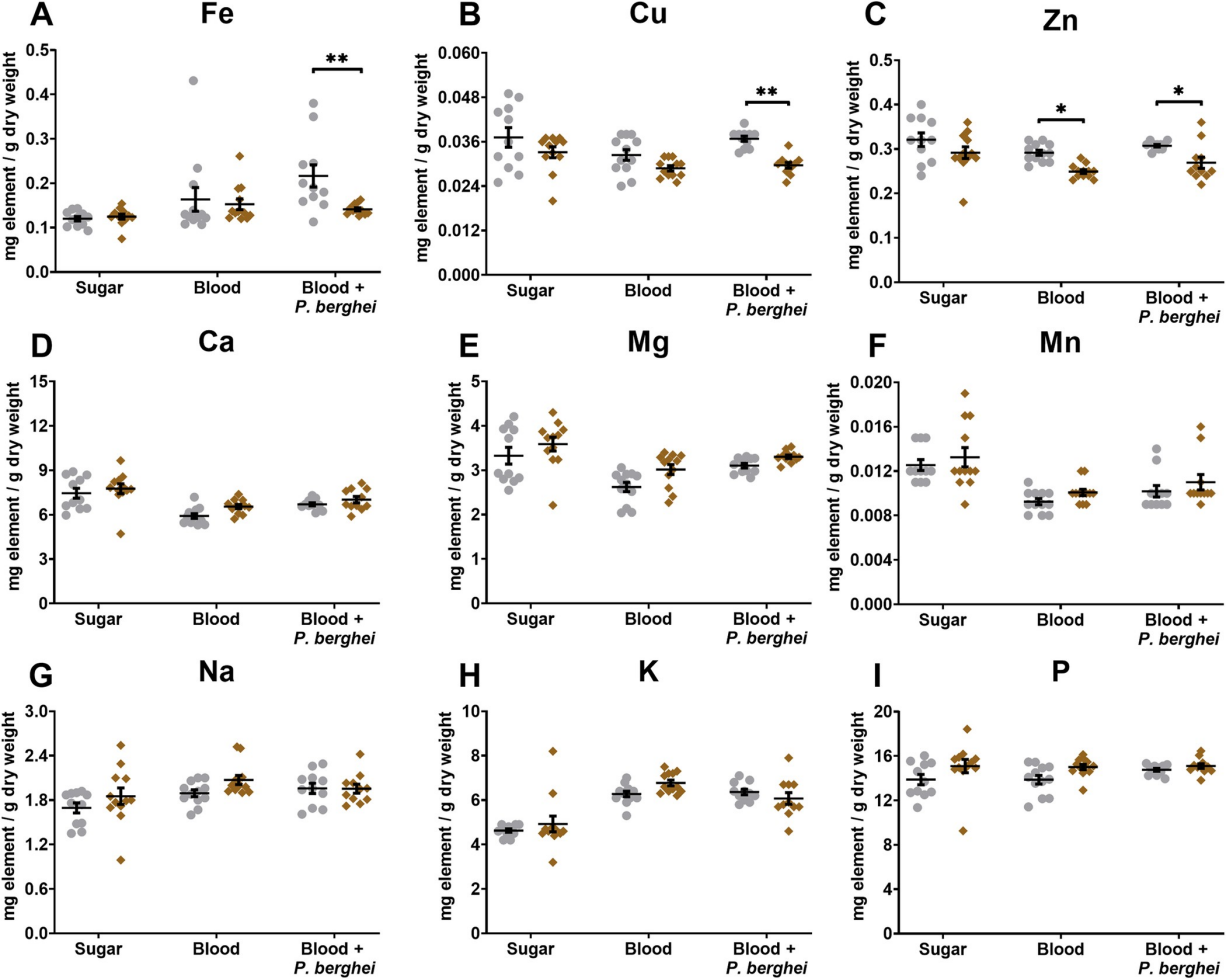
Metallomics following *P*. *berghei* infection of *An*. *albimanus ws* and *bs* phenotypes. Elemental concentrations of A) iron, B) copper, C) zinc, D) calcium, E) magnesium, F) manganese, G) sodium, H) potassium, and I) phosphorus, were determined in *ws* (grey circles) and *bs* (brown diamonds) phenotypes from *An*. *albimanus* seven days post *P*. *berghei* infection (Blood + *P*. *berghei*), post feeding with uninfected blood (Blood) or continued feeding with sugar only (Sugar). The data are expressed in mg element per g dry weight, showing the mean ± SE from independent measurements taken in three experimental repeats. Numerical values can be found in S3 Table. Two-way ANOVA followed by Bonferroni’s multiple comparisons test was used to determine differences between phenotypes in each treatment; indicated with two asterisks. Specifically, ** for Fe *p* = 0.006, ** for Cu *p* = 0.003, * for Zn (Blood) *p* = 0.01, * for Zn (Blood + *P*. *berghei*) *p* = 0.03.

To assess whether infection with *P*. *berghei* alters the metallome of the *ws* and *bs* phenotypes from *An*. *albimanus*, metal measurements in female mosquitoes fed with *P*. *berghei*-infected blood, uninfected blood, and sugar solution were performed seven days post-blood (±infection) feeding and same age mosquitoes for the sugar-fed group (Fig 3). At this time point, blood digestion and excretion had been completed. Previously observed differences in infection susceptibility between the *ws* and *bs* phenotypes [56,59] were re-examined and confirmed (S2 Fig). Median infection, represented as the number of *P*. *berghei* oocysts per midgut, was of six in the susceptible *ws* phenotype, compared to four and a half for the resistant *bs* phenotype. We reasoned that potential differences in the manner metal ions accumulate in response to infection might be associated with the two phenotypes’ differential resistance to *P*. *berghei*. We therefore performed elemental analysis on all groups of mosquitoes (Fig 3).

Twelve-day-old female mosquitoes of the two phenotypes raised on sugar medium showed no significant difference between *ws* and *bs* for any of the elements assessed (Fig 3). The mean value for zinc was 16% higher for *ws versus bs* when feeding on blood and after allowing for sufficient time to digest and excrete the meal (Fig 3C). Except for zinc, the concentration of other metal ions was affected similarly between the two phenotypes after the blood meal (S3 Table). Specifically, considering pooled data from both *An*. *albimanus* phenotypes, blood-feeding led to a decrease in the concentration of calcium (-18%), magnesium (-19%), and manganese (-25%), and to an increase of potassium (+36%). Phosphorus was the only measured element that showed no change upon blood feeding (Fig 3I). Less robust changes in zinc (-12%), copper (-13%), iron (+24%) and sodium (+12%) were driven largely from one of the two phenotypes as can be assessed from the detailed statistical analysis (S3 Table).

Next, we compared the metallomes of *ws* and *bs* mosquitoes between blood-fed and infected groups (Fig 3 and S3 Table). In the resistant *bs* phenotype, no differences were observed for any metal ion associated with the presence of infection. Intriguingly, in the susceptible *ws* phenotype, iron increased further upon infection by 32% (Fig 3A), and copper increased by 14% (Fig 3B). Iron and copper were the only metal ions that showed a different response between the two phenotypes.

### The effects of iron and copper chelation on the outcome of *P*. *berghei* infections and follicular development

**Fig 4:**
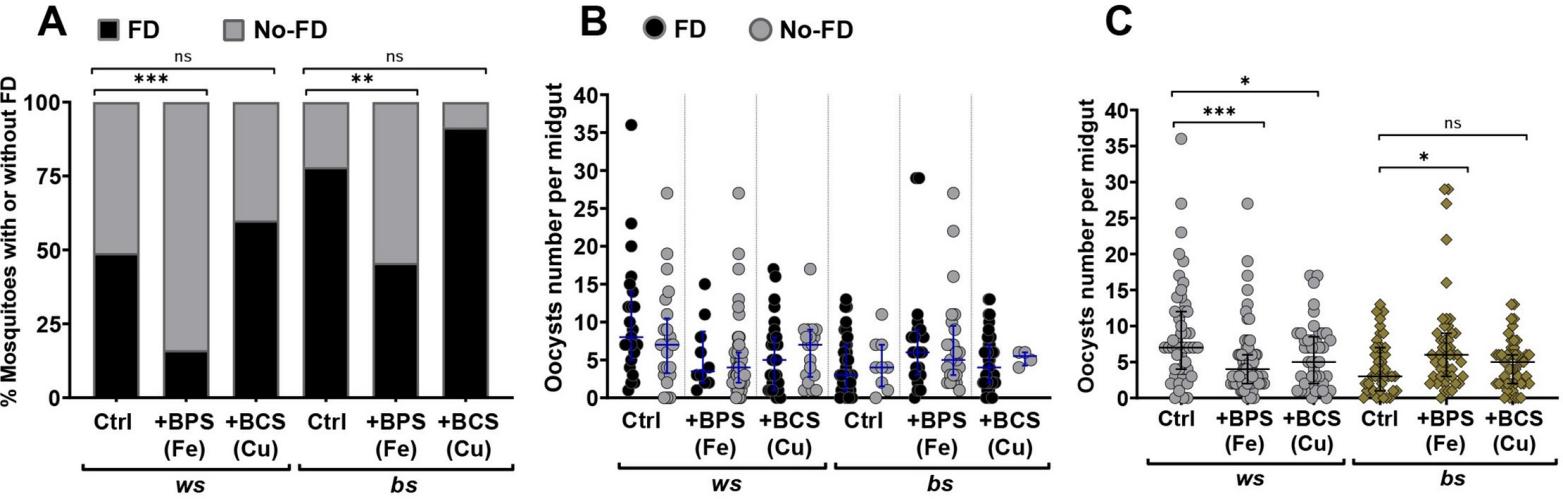
Effect of iron and copper chelation on follicular development and *P*. *berghei* infection in *ws* and *bs An*. *albimanus*. Mosquitoes were fed with sugar solution (Ctrl) or sugar solution supplemented with BPS or BCS throughout their adult life. Four days-old mosquitoes were fed with *P*. *berghei*-infected blood. A) Percentage of mosquitoes with follicular development (FD) (in black bars), and without follicular development (No-FD) (in grey bars) was determined in each treatment group. BPS treatment further suppressed oogenesis in both *ws* and *bs* mosquitoes. Data were analyzed using chi-squared test, statistical differences are indicated by asterisks, ** *p =* 0.002 (n = 46; X^2^ = 9.6) and *** *p =* 0.0002 (n = 62; X^2^ = 13.6), ns = non-significant (*p>0*.*05*). B) Oocyst number was determined seven days post-infection and plotted against the presence or absence of FD, within treatment groups. Circles in black represent individuals with FD, circles in grey correspond to individuals with No-FD. Medians with interquartile range are indicated in blue. No statistical differences (*p>0*.*05*) were found using Mann-Whitney U test between FD and No-FD conditions in each group. C) The same data as in the previous panel was pooled together for statistical analysis with Mann-Whitney U test comparing with control group inside each phenotype. Statistical differences are indicated by asterisks, * *p =* 0.0138, *** *p =* 0.0004 for *ws* and * *p =* 0.0258, ns = *p*>0.05 for *bs* comparisons between BPS and BCS, respectively, and control treatments. Medians with interquartile range are indicated in black. Infection distribution within each phenotype is described in the S4 Table.

We decided to explore the role of iron and copper during *P*. *berghei* infection in both phenotypes. This was approached by adding to the sugar solution 200 μM bathophenanthroline sulfate (BPS) or 200 μM bathocuproine sulfate (BCS) from the emergence of the mosquitoes until the day of oocyst counting (same timeline as above) to chelate iron and copper, respectively. Mosquitoes were fed on *P*. *berghei* infected blood on day five of adulthood as in the previous experiments. These metal chelators have been used before in *D*. *melanogaster* at dietary concentrations between 100 and 300 μM, acting with proven metal specificities [74,75]. As previously shown for some *Anopheles* species [76–78], *P*. *berghei* infection affected follicular development in 51% (n = 47) of the susceptible *ws* female mosquitoes and 22% (n = 41) of the resistant *bs* counterparts (Fig 4). Application of BPS enhanced the inhibitory effect on follicular development in both phenotypes taking the percentages to 84% (n = 62; X^2^ = 13.6, *p =* 0.0002) and 54% (n = 46; X^2^ = 9.6; *p =* 0.002), respectively. This result is consistent with the previously shown requirement of iron for oogenesis in *Ae*. *aegypti* [24,25]. Each individual female either had fully-developed or immature ovaries, meaning that the response was of an "all-or-none" type (S3 Fig). In contrast to iron, copper availability did not alter follicular development in response to infection, as judged from the application of BCS to either phenotype (Fig 4A; *p* = 0.287 for *ws* and *p* = 0.076 for *bs* comparisons, respectively).

Previous studies have suggested trade-offs between reproductive fitness and immune defense in *An*. *gambiae* [76], reproduction and survival in *C*. *pipiens* [77], parasite elimination and egg production in *An*. *albimanus* [78]. We wondered whether there was an association between the number of *P*. *berghei* oocysts colonizing the *An*. *albimanus* midgut and the inhibition of FD. Therefore, we compared infection intensity considering the presence or absence of FD in each mosquito group (Fig 4B). No significant differences were found between infection in mosquitoes with or without FD within each treatment group. Nevertheless, application of either BPS or BCS significantly reduced *P*. *berghei* infection in the otherwise susceptible *ws* phenotype (Fig 4C). Specifically, a median infection of seven oocysts per midgut in the control group was reduced to four with the iron chelator (*p* = 0.0004) and five with the copper chelator (*p* = 0.01). The *bs* phenotype under control conditions showed a median infection of three and no statistically significant difference to the BCS-treated *bs* mosquitoes (median of five). Surprisingly, BPS-treated *bs* mosquitoes showed a median value of six (*p* = 0.03), reversing the normally resistant *bs* phenotype into a susceptible condition to infection (Fig 4C and S4 Table).

**Fig 5:**
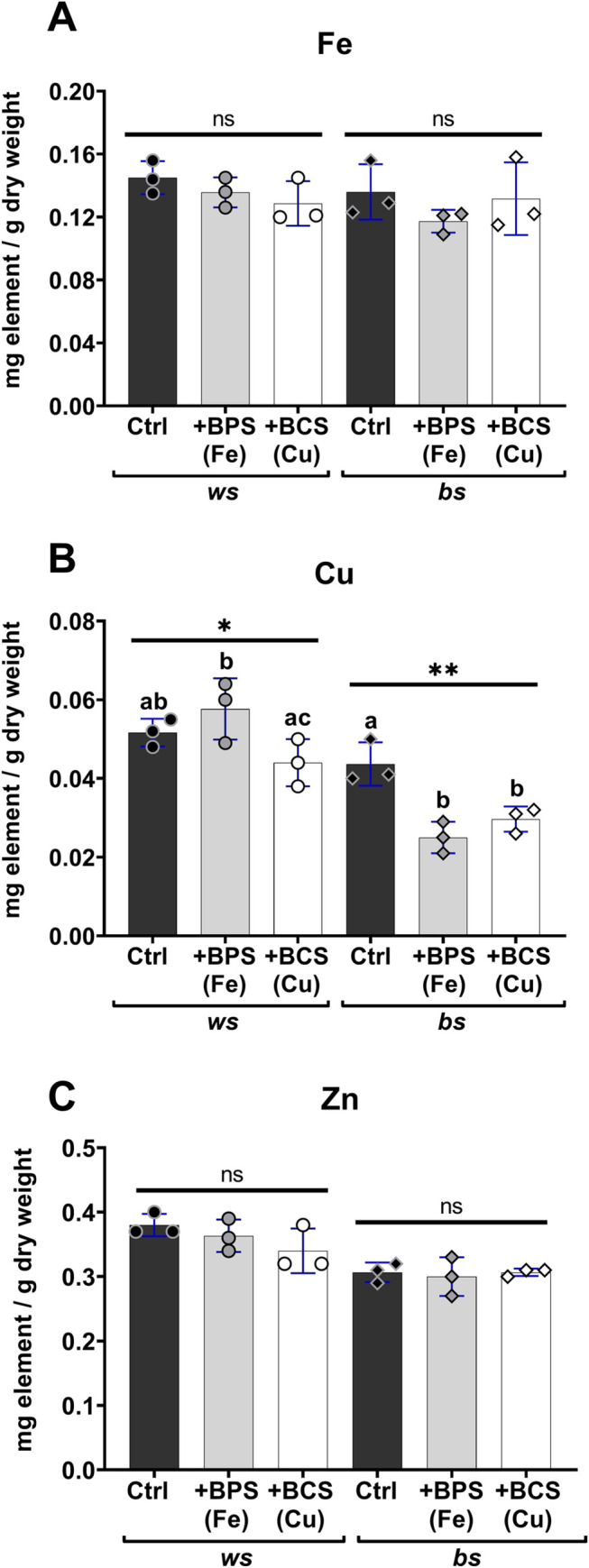
Metal concentrations in *P*. *berghei*-infected *ws* and *bs* mosquitoes treated additionally with metal chelators. At seven days post-infection with *P*. *berghei* elemental analysis was performed on adult female mosquitoes as indicated. For both *ws* and *bs* phenotypes, Ctrl (darker bars) indicates infected group without metal chelator treatment, BPS (grey bars) indicates the effects of dietary iron chelation, while BCS (white bars) is for copper chelation. Three independent measurements were done (circles for *ws* and diamonds for *bs*) and SD is represented in blue. Data were analyzed with one-way ANOVA followed by Tukey’s test. Statistical differences between groups are indicated by different letters, and asterisks denote *p* values as follows: * *p* = 0.0370, ** *p* = 0.0055, ns = *p*>0.05.

To assess the metal chelators’ effects in the mosquitoes’ whole-body metallome, we performed the elemental analysis at the experiment’s endpoint (Fig 5). The corresponding effects of BPS on whole-body iron and of BCS on whole-body copper were negligible in the *ws* mosquitoes (mean values were 11% and 14% lower for iron and copper, respectively, but these reductions were not statistically significant). BCS had a clear and specific effect lowering copper accumulation by 33% in the *bs* phenotype. Although iron was not statistically significant different in *bs* mosquitoes fed with BPS, this treatment unexpectedly decreased copper by 43% (Fig 5B). Except for this last finding, which appears to be associated with a particularity of metal metabolism in the *bs* phenotype, the metal chelators worked according to their known metal specificities. In keeping with this conclusion, zinc concentration in the mosquitoes was unaffected during the various treatments (Fig 5C).

## Discussion

### Metal profiles differ during the development of mosquitoes

Previous studies of how insect metallomes vary with respect to their evolutionary histories and specific ecology suggested that transition metal ions accumulate with the enzyme systems that require them as cofactors; variation amongst species was mostly attributable to changes in storage mechanisms or differing diets [79,80]. Hematophagous insects are of particular interest for the latter parameter. Additionally, as this study demonstrates, it is essential to consider the different developmental stages (Fig 2). Mosquito larvae grow in an aquatic environment, in contrast to the terrestrial life of adults. Higher sodium and potassium concentration may relate to adaptations required for living in a hypo-osmotic condition [81–83]. A comment is due regarding the considerable variability reported for the *Ae*. *aegypti* NO strain (Fig 2); in contrast to the other three, this laboratory-reared strain was studied on four separate occasions at different seasons and years. The corresponding data points cluster as per the independent replicates from each sample collection. The variability cannot be attributed to measurement issues because *D*. *melanogaster* standards were run in parallel showing no difference between experiments. It is unclear if the variability could be attributed to different food batches or seasonal fluctuations (uncontrolled variables) or inherent biological variance between generations [47,48]. The results serve as a point of caution for future studies as they suggest a range of plasticity in metal concentrations, especially during the larval stages of development. For these reasons, the comparison between the Rock and NO strains presented here should be considered of preliminary nature. Given the proposed role of iron in dengue infection [49–51], similar studies as the one we performed here for *Plasmodium* infection in *Anopheles* are called for. Further work with different mosquito species would also contribute to generalizations or identifying species-specific patterns in mosquito metallomics. Overall, the data reported here suggest similar metallome patterns between *An*. *albimanus* and *Ae*. *aegypti* with only few exceptions that were already described in detail above.

### Susceptibility to *P*. *berghei* infection in the *ws* phenotype was associated with increased iron and copper accumulation in the mosquitoes

The genetic and physiological reasons that underlie the different susceptibility to *P*. *berghei* infection between *ws* and *bs* phenotypes are unknown [56–59]. Previous work looking at resistance of *An*. *gambie* to *P*. *bergei* transmission identified the complement-like protein TEP1 interacting with the leucine-rich protein complex LRIM1 and APL1C for the parasite’s killing, followed by parasite elimination at a later step with melanization reactions under the control of NFkB signaling [84–86]. Interestingly, TEP1, LRIM1 and other immune genes were indeed expressed at higher level in the resistant *bs* strain, whereas melanization genes, such as PPO1, showed reduced expression [59]. The latter finding is consistent with a general tendency for degree of melanization reactions correlating with iron and copper concentrations [87], as observed here in the comparison between *ws* and *bs* phenotypes (discussed in the following paragraph). Of interest in respect to melanization is a recent discovery of a conserved transporter gene, which turns pupae of Tephritidae flies white when mutated [88].

We noted the following differences between the metallomes of the two phenotypes of *An*. *albimanus*. First, there is a moderate (10–30%) increase in zinc concentration in *ws* adult mosquitoes than similarly grown and aged *bs* counterparts (Figs 2C, 3C and 5C). Although this difference in zinc is independent of the presence or absence of *P*. *berghei*, it may be related with increased expression of TEP1 and other immune genes in the *bs* mosquitoes [59], given the association of zinc with proteins of the mammalian complement system [89,90]. Second, we noted that *ws* third instar larvae accumulate several metal ions during the larval developmental stage, where the characteristic white stripe first appears. Iron and copper show significant differences compared to *bs* larvae (Fig 2A and 2B). The underlying reason for these differences has not been explored, however, iron and copper also accumulate specifically in infected adult females of the *ws* phenotype (Fig 3A and 3B). We tested whether dietary supplementation with a metal chelator might render the *ws* phenotype more resistant to *P*. *berghei* infection and found that it did (Fig 4C). These results are in line with the simple interpretation that, as shown for its stages of development in the mammalian host [62–73], *Plasmodium* also requires access to iron and copper in the mosquito.

### The unexpected reversal of resistance to *P*. *berghei* infection in the *bs* phenotype by iron chelation

Turning to the *bs* phenotype, which was resistant to *P*. *berghei* infection to start with, BCS treatment had no further effect. This finding is still consistent with the idea that the original resistance to *Plasmodium* in the *bs* phenotype might be associated with robust metal-withdrawing strategies. However, these interpretations were challenged by the finding that BPS treatment rendered *bs* mosquitoes susceptible to infection (Fig 4C). BPS treatment is not expected to affect copper accumulation (see, for example, BPS treatment of the *ws* phenotype here in Fig 5B, or reference [74]). However, it reduced copper by 43% in *bs* mosquitoes. The interaction between copper and iron metabolism in insects remains under investigation [32–36,87]. Since *bs* mosquitoes showed a lower concentration of iron and copper, the increase in susceptibility after the BPS treatment, which further lowered the concentration of these metals, may be due to a detrimental deficiency of metal ions as prosthetic groups in proteins responsible for eliminating the parasite. Although we cannot explain the particular response of *bs* phenotype mosquitoes to the iron chelator, the results collectively suggest that both iron and copper are involved in the mosquito’s interaction with *Plasmodium*. Further work in this area is needed to uncover the mechanistic details of both interactions, taking into account the limitations of whole body elemental determinations, which do not provide information on tissue-specific and subcellular distribution and bioavailability of the metal ions.

### Follicular development did not correlate with the severity of *P*. *berghei* infection

The observation that *P*. *berghei* infection affected oogenesis is consistent with previous reports [76–78]. Likewise, iron chelation’s additive inhibitory effect on oogenesis observed in both *ws* and *bs* phenotypes were not unexpected given what is known for the role of iron in oogenesis in *Ae*. *aegypti* [24,25]. Two further findings are presented here. First, copper chelation had no inhibitory effect on oogenesis (Fig 4A), suggesting different dietary requirements for these two metals in reproduction. Second, we were surprised to find no relation between female mosquitoes that suppressed oogenesis and infection severity (Fig 4B). This result should inform ongoing research over trade-offs between the insect’s reproductive ability and its response to infection [76–78].

### Interference with mosquito metal ion homeostasis is an unexplored area in vector control programs

Malaria remains a deadly disease, which is best combatted by controlling mosquito populations [61,62]. A variety of approaches are currently being undertaken with this goal [1–8]. We propose that metal chelators offer an additional tool that is worth considering in vector-borne disease control programs and potentially also in the control or monitoring of fruit flies of economic impotance [80]. Further work in this area should also study chelator application during larval development, when more substantial effects may be expected given the requirement of metal ions for insect growth [9,13].

## Methods

### Mosquito rearing

The *ws* and *bs* colonies of the *An*. *albimanus* Tapachula strain were reared at insectary conditions of 28 ± 1°C, humidity at 80% and 12 hours photoperiod. Larval stages were fed with cat food (chicken, cereals, and milk mix) for 2–12 months kitten (Whiskas) [91]. The *ws* and *bs* phenotypes have been reared independently for more than sixty generations resulting in stable colonies (S1 Fig). Adult mosquitoes were fed with 10% sugar solution using soaked cotton pads. For *Anopheles* mosquitoes, the sugar solution was supplied with 4-Aminobenzoic acid (PABA) 0.05% and antibiotic-antimicotic 1X (GIBCO). The *An*. *albimanus* colonies and the *Ae*. *aegypti* Rockefeller strain were reared at the insectary of the National Institute of Public Health (INSP) in Cuernavaca, Morelos, México. The *Ae*. *aegypti* New Orleans strain was reared at the insectary of the Collaborative Unit for Entomological Bioassays (UCBE) of the Autonomous University of Yucatán (UADY) in Mérida, Yucatán, México. Larval stages were fed with a meat flour and yeast (8:2) solution. Adult mosquitoes were fed with 10% sugar solution using a felt strip device. The metal ion content of the two diets was determined (S2 Table).

### Parasite culturing

The clone 2.34 of the *Plasmodium berghei* ANKA strain, which constitutively expresses the green fluorescent protein [92], was used in all infection experiments. Ookinete culture was performed according to a previously published protocol [93]. Briefly, blood-stage parasites were kept in liquid nitrogen and inoculated intraperitoneally (IP) in six to eight weeks-old male BALB/c mice. Parasitaemia was determined by Giemsa smears, 10^5^ parasites were propagated IP up to the eighth pass. For ookinete culture, the mice were treated with 200 μl of phenylhydrazine at 6 mg/ml three days before the inoculation of 5 x 10^7^ parasites. Three days post-inoculation the infected blood was extracted via cardiac puncture of a CO_2_ euthanized mice, using a heparinized syringe (250 IU of heparin / ml of blood). Blood was diluted 1:5 in ookinete medium (RPMI 1640 medium at pH 8.3 supplemented with 23.81 mM sodium bicarbonate, 0.37 mM hypoxanthine, 25 mM HEPES, 5000 U/ml penicillin, 5mg/ml streptomycin, 10 mg/ml neomycin and 20% heat-inactivated fetal bovine serum) and incubated for 18 h at 20°C to allow ookinete formation. Only mice with a parasitaemia between 15 to 25% and with more than 7 exflagellation centers in a 400X field were used. The exflagellation was evaluated prior to mouse sacrifice by diluting tail-blood in ookinete medium (1:4), incubated at 20°C for 15 minutes.

### *Plasmodium* infection

For infection, four days-old mosquitoes were fed with 8 x 10^5^ ookinetes per ml in all experiments. Ookinetes were centrifuged at 1500g and re-suspended using blood of an uninfected mouse. After feeding, mosquitoes were kept at 21°C to permit *Plasmodium* development. Seven days after feeding, mosquitoes were dissected in phosphate buffered saline; midguts were collocated on slides over RPMI medium drops to permit oocyst counting using an epi-fluorescence microscopy (Leica DM 1000). Additionally, during midgut dissections, we obtained ovaries to determine the presence or absence of follicular development, as was previously reported in *An*. *albimanus* during *P*. *berghei* infection [78].

### Treatment with chelators

Bathophenanthrolinedisulfonic acid disodium salt hydrate (BPS; Sigma-Aldrich #146617) and bathocuproinedisulfonic acid disodium salt (BCS; Sigma-Aldrich #B1125) were used as extracellular iron and copper chelators, respectively [73,74]. Chelators were added to 10% sugar solution at a concentration of 200 μM and were administrated using soaked cotton pads throughout the adult lifespan of the mosquitoes, *i*.*e*. for five days prior to and seven days after the *Plasmodium* infected blood meal.

### Sample collection

For mosquito development assays, samples from *Anopheles* and *Aedes* mosquitoes were collected at different larvae instars. Three days-old male and female mosquitoes feeding exclusively on 10% sugar solution were used for the adult stages. Each sample was collected and dried in a precipitate vase glass (5 ml) during 72 hours at 50°C in order to remove water. Dried samples were collected using a flat spatula, weighed and placed in Eppendorf tubes. For the *Plasmodium* infection assays, mosquitoes were collected at day seven post-treatment. Each sample consisted of 30 females dried in a glass Petri dish and treated as explained above.

### Elemental analysis

Inductively coupled plasma optic emission spectrometry (ICP-OES) was used for metal determinations in all samples. Between 10 and 20 mg of dry sample was digested with 1 mL of nitric acid at 200°C for 15 min in a MARS6 microwave digestion system (NC, USA). Samples were adjusted to 5mL volume with water. Total metal concentrations were measured against a calibration curve in the PerkinElmer Optima 8300 ICP-OES (CT, USA) instrument and presented as mg metal per g sample dry weight.

### Statistical analysis

All data were analyzed in GraphPad Prism version 8. Two-way ANOVA (phenotype/strain *versus* instar/stage) was used for analysis of the metallomics results during development (Fig 2 and S1 Table), after first checking normality distributions of the data with the Shapiro-Wilk test. Bonferroni’s multiple comparisons test was used to determine statistical differences between phenotypes or strains in the same instar/stage, and Tukey’s test was used to determine differences between instar/stage within the same phenotype or strain. Two-way ANOVA was also used when considering phenotype (*ws* or *bs*) and treatment (sugar, blood or blood + *P*. *berghei*) as variables for the analysis of the metallomes after blood feeding and infection (Fig 3 and S3 Table). Bonferroni’s multiple comparisons test was used to determine statistical differences in each treatment between phenotypes (*ws versus bs*) and Tukey’s test was used to determine differences between treatments in the same phenotype (sugar *versus* blood, sugar *versus* blood + *P*. *berghei*, and blood *versus* blood + *P*. *berghei*). The chi-squared test was used to determine the probability of follicular development between groups treated with chelators and control groups (Fig 4A). The Mann-Whitney U test was used to determined statistical differences between medians in infected groups (Figs 4B, 4C and S2 and S4 Table). One-way ANOVA followed by Tukey’s test was used to determine differences after chelator treatments (Fig 5).

## Supporting information

S1 Fig Phenotypes of *An. albimanus* Tapachula strain. The parental strain contains both *ws* and *bs* phenotypes (top image). Following inbreeding for over sixty generations, the two phenotypes have been successfully separated (lower images).
(TIF)

S2 Fig *P. berghei* infection *in ws* and *bs* mosquitoes *An. albimanus*. Oocyst number per midgut of *ws* (grey circles) and *bs* (brown diamonds) mosquitoes were determined at seven days post-feeding with *P*. *berghei*-infected blood. Data were analyzed with the Mann-Whitney U test, statistical difference is indicated by asterisks, ***p = 0.0006. Medians with interquartile range are indicated in black. Infection distributions for each phenotype are described in the table.
(TIF)

S3 Fig Blood feeding triggers follicular development (oogenesis). Dissected ovaries from *ws* (left) and *bs* (right) phenotypes of *An*. *albimanus*. After blood-feeding, ovarian development was observed in both phenotypes. Ovaries from blood-fed (BF) or sugar-fed (SF) mosquitoes are indicated with arrows. Scale bar = 500 μm.
(TIF)

S1 Table Elemental analysis of *Ae. aegypti* and *An. albimanus* throughout their life cycle. (XLSX)

S2 Table Elemental analysis of larval diets used in this study. Results are given in mg element per g of diet. The diet used for NO was also used for rearing of *An*. *albimanus* larvae.
(DOCX)

S3 Table Elemental analysis of adult female mosquitoes at seven days after they were fed on infected and uninfected blood. (XLSX)

S4 Table Infection distribution at seven days post *P. berghei* infection of *ws* and *bs* phenotypes co-subjected to chelator treatments. (DOCX)

## References

[1] Manson P. On the development of Filaria sanguis hominis and on the mosquito considered as a nurse. J Linn Soc (Zool). 1878;14:304–311.

[2] Ferguson NM. Challenges and opportunities in controlling mosquito-borne infections. Nature. 2018;559:490–497. doi: 10.1038/s41586-018-0318-5 30046071

[3] Adolfi A, Lycett GJ. Opening the toolkit for genetic analysis and control of Anopheles mosquito vectors. Curr Opin Insect Sci. 2018;30:8–18. doi: 10.1016/j.cois.2018.07.014 30553490

[4] Hoermann A, Tapanelli S, Capriotti P, Del Corsano G, Masters EK, Habtewold T, et al. Converting endogenous genes of the malaria mosquito into simple non-autonomous gene drives for population replacement. Elife. 2021;10:e58791. doi: 10.7554/eLife.58791 33845943 PMC8043746

[5] Gomes FM, Barillas-Mury C. Infection of anopheline mosquitoes with Wolbachia: Implications for malaria control. PLoS Pathog. 2018;14:e1007333. doi: 10.1371/journal.ppat.1007333 30440032 PMC6237385

[6] Barreaux P, Barreaux AMG, Sternberg ED, Suh E, Waite JL, Whitehead SA, Thomas MB. Priorities for Broadening the Malaria Vector Control Tool Kit. Trends Parasitol. 2017;33:763–774. doi: 10.1016/j.pt.2017.06.003 28668377 PMC5623623

[7] Rivera-Pérez C, Clifton ME, Noriega FG. How micronutrients influence the physiology of mosquitoes. Curr Opin Insect Sci. 2017;23:112–117. doi: 10.1016/j.cois.2017.07.002 29129275 PMC5695569

[8] Samaddar S, Marnin L, Butler LR, Pedra JHF. Immunometabolism in Arthropod Vectors: Redefining Interspecies Relationships. Trends Parasitol. 2020;36:807–815. doi: 10.1016/j.pt.2020.07.010 32819827 PMC7897511

[9] Marelja Z, Leimkühler S, Missirlis F. Iron Sulfur and Molybdenum Cofactor Enzymes Regulate the Drosophila Life Cycle by Controlling Cell Metabolism. Front Physiol. 2018;9:50. doi: 10.3389/fphys.2018.00050 29491838 PMC5817353

[10] Navarro JA, Schneuwly S. Copper and Zinc Homeostasis: Lessons from Drosophila melanogaster. Front Genet. 2017;8:223. doi: 10.3389/fgene.2017.00223 29312444 PMC5743009

[11] Dow JA. The essential roles of metal ions in insect homeostasis and physiology. Curr Opin Insect Sci. 2017;23:43–50. doi: 10.1016/j.cois.2017.07.001 29129281

[12] Calap-Quintana P, González-Fernández J, Sebastiá-Ortega N, Llorens JV, Moltó MD. Drosophila melanogaster Models of Metal-Related Human Diseases and Metal Toxicity. Int J Mol Sci. 2017;18:1456. doi: 10.3390/ijms18071456 28684721 PMC5535947

[13] Missirlis F. Regulation and biological function of metal ions in Drosophila. Curr Opin Insect Sci. 2021;47:18–24. doi: 10.1016/j.cois.2021.02.002 33581350

[14] Whiten SR, Eggleston H, Adelman ZN. Ironing out the Details: Exploring the Role of Iron and Heme in Blood-Sucking Arthropods. Front Physiol. 2018;8:1134. doi: 10.3389/fphys.2017.01134 29387018 PMC5776124

[15] Dunkov BC, Zhang D, Choumarov K, Winzerling JJ, Law JH. Isolation and characterization of mosquito ferritin and cloning of a cDNA that encodes one subunit. Arch Insect Biochem Physiol. 1995;29:293–307. doi: 10.1002/arch.940290307 7655055

[16] Yoshiga T, Hernandez VP, Fallon AM, Law JH. Mosquito transferrin, an acute-phase protein that is up-regulated upon infection. Proc Natl Acad Sci U S A. 1997;94:12337–42. doi: 10.1073/pnas.94.23.12337 9356450 PMC24933

[17] Zhang D, Dimopoulos G, Wolf A, Miñana B, Kafatos FC, Winzerling JJ. Cloning and molecular characterization of two mosquito iron regulatory proteins. Insect Biochem Mol Biol. 2002;32:579–89. doi: 10.1016/s0965-1748(01)00138-2 11891134

[18] Tan W, Wang X, Cheng P, Liu L, Wang H, Gong M, et al. Molecular cloning and preliminary function study of iron responsive element binding protein 1 gene from cypermethrin-resistant Culex pipiens pallens. Parasit Vectors. 2011;4:215. doi: 10.1186/1756-3305-4-215 22075242 PMC3223502

[19] Martínez-Barnetche J, García Solache M, Neri Lecona A, Tello López AT, del Carmen Rodríguez M, Gamba G, et al. Cloning and functional characterization of the Anopheles albimanus DMT1/NRAMP homolog: implications in iron metabolism in mosquitoes. Insect Biochem Mol Biol. 2007;37:532–9. doi: 10.1016/j.ibmb.2007.02.009 17517330

[20] Tsujimoto H, Anderson MAE, Myles KM, Adelman ZN. Identification of Candidate Iron Transporters From the ZIP/ZnT Gene Families in the Mosquito Aedes aegypti. Front Physiol. 2018;9:380. doi: 10.3389/fphys.2018.00380 29706902 PMC5906682

[21] Tsujimoto H, Anderson MAE, Eggleston H, Myles KM, Adelman ZN. Aedes aegypti dyspepsia encodes a novel member of the SLC16 family of transporters and is critical for reproductive fitness. PLoS Negl Trop Dis. 2021;15:e0009334. doi: 10.1371/journal.pntd.0009334 33826624 PMC8055033

[22] Spencer CS, Yunta C, de Lima GPG, Hemmings K, Lian LY, Lycett G, et al. Characterisation of Anopheles gambiae heme oxygenase and metalloporphyrin feeding suggests a potential role in reproduction. Insect Biochem Mol Biol. 2018;98:25–33. doi: 10.1016/j.ibmb.2018.04.010 29729387

[23] Bottino-Rojas V, Pereira LOR, Silva G, Talyuli OAC, Dunkov BC, Oliveira PL, et al. Non-canonical transcriptional regulation of heme oxygenase in Aedes aegypti. Sci Rep. 2019;9:13726. doi: 10.1038/s41598-019-49396-3 31551499 PMC6760526

[24] Zhou G, Kohlhepp P, Geiser D, Frasquillo Mdel C, Vazquez-Moreno L, Winzerling JJ. Fate of blood meal iron in mosquitoes. J Insect Physiol. 2007;53:1169–78. doi: 10.1016/j.jinsphys.2007.06.009 17689557 PMC2329577

[25] Geiser DL, Thai TN, Love MB, Winzerling JJ. Iron and Ferritin Deposition in the Ovarian Tissues of the Yellow Fever Mosquito (Diptera: Culicidae). J Insect Sci. 2019;19:11. doi: 10.1093/jisesa/iez089 31606748 PMC6790249

[26] Kremer N, Voronin D, Charif D, Mavingui P, Mollereau B, Vavre F. Wolbachia interferes with ferritin expression and iron metabolism in insects. PLoS Pathog. 2009;5:e1000630. doi: 10.1371/journal.ppat.1000630 19851452 PMC2759286

[27] Kosmidis S, Missirlis F, Botella JA, Schneuwly S, Rouault TA, Skoulakis EM. Behavioral decline and premature lethality upon pan-neuronal ferritin overexpression in Drosophila infected with a virulent form of Wolbachia. Front Pharmacol. 2014;5:66. doi: 10.3389/fphar.2014.00066 24772084 PMC3983519

[28] Lima VL, Dias F, Nunes RD, Pereira LO, Santos TS, Chiarini LB, et al. The antioxidant role of xanthurenic acid in the Aedes aegypti midgut during digestion of a blood meal. PLoS One. 2012;7:e38349. doi: 10.1371/journal.pone.0038349 22701629 PMC3372515

[29] Jiang Y, Wei J, Cui H, Liu C, Zhi Y, Jiang Z, et al. An intracellular membrane protein GEP1 regulates xanthurenic acid induced gametogenesis of malaria parasites. Nat Commun. 2020;11:1764. doi: 10.1038/s41467-020-15479-3 32273496 PMC7145802

[30] Mandilaras K, Pathmanathan T, Missirlis F. Iron absorption in Drosophila melanogaster. Nutrients. 2013;5:1622–47. doi: 10.3390/nu5051622 23686013 PMC3708341

[31] Eggleston H, Adelman ZN. Transcriptomic analyses of Aedes aegypti cultured cells and ex vivo midguts in response to an excess or deficiency of heme: a quest for transcriptionally-regulated heme transporters. BMC Genomics. 2020;21:604. doi: 10.1186/s12864-020-06981-5 32867680 PMC7460771

[32] Gorman MJ, Dittmer NT, Marshall JL, Kanost MR. Characterization of the multicopper oxidase gene family in Anopheles gambiae. Insect Biochem Mol Biol. 2008;38:817–24. doi: 10.1016/j.ibmb.2008.07.001 18675911 PMC2573861

[33] Peng Z, Dittmer NT, Lang M, Brummett LM, Braun CL, Davis LC, et al. Multicopper oxidase-1 orthologs from diverse insect species have ascorbate oxidase activity. Insect Biochem Mol Biol. 2015;59:58–71. doi: 10.1016/j.ibmb.2015.02.005 25701385 PMC4387078

[34] Bettedi L, Aslam MF, Szular J, Mandilaras K, Missirlis F. Iron depletion in the intestines of Malvolio mutant flies does not occur in the absence of a multicopper oxidase. J Exp Biol. 2011;214:971–8. doi: 10.1242/jeb.051664 21346125

[35] Lang M, Braun CL, Kanost MR, Gorman MJ. Multicopper oxidase-1 is a ferroxidase essential for iron homeostasis in Drosophila melanogaster. Proc Natl Acad Sci U S A. 2012;109:13337–42. doi: 10.1073/pnas.1208703109 22847425 PMC3421175

[36] Wang X, Yin S, Yang Z, Zhou B. Drosophila multicopper oxidase 3 is a potential ferroxidase involved in iron homeostasis. Biochim Biophys Acta Gen Subj. 2018;1862:1826–1834. doi: 10.1016/j.bbagen.2018.04.017 29684424

[37] Sarkar S, Duttagupta AK, Mal TK. Effects of heavy metals on population growth and metallothionein gene expression in the mosquito Culex quinquefasciatus, from Calcutta, India. Environ Pollut. 2004;127:183–93. doi: 10.1016/j.envpol.2003.08.005 14568718

[38] Mireji PO, Keating J, Hassanali A, Impoinvil DE, Mbogo CM, Muturi MN, et al. Expression of metallothionein and alpha-tubulin in heavy metal-tolerant Anopheles gambiae sensu stricto (Diptera: Culicidae). Ecotoxicol Environ Saf. 2010;73:46–50. doi: 10.1016/j.ecoenv.2009.08.004 19735939 PMC2783303

[39] Perez MH, Noriega FG. Sub-lethal metal stress response of larvae of Aedes aegypti. Physiol Entomol. 2014;39:111–119. doi: 10.1111/phen.12054 24926118 PMC4049351

[40] Doty AH. The Use of Sulphate of Copper Alone, and in Combination with Lime for the Destruction of Mosquito Larvae-as a Deodorant, and as a Disinfectant. Public Health Pap Rep. 1905;30:50–7. 19601187 PMC2222332

[41] Rayms-Keller A, Olson KE, McGaw M, Oray C, Carlson JO, Beaty BJ. Effect of heavy metals on Aedes aegypti (Diptera: Culicidae) larvae. Ecotoxicol Environ Saf. 1998;39:41–7. doi: 10.1006/eesa.1997.1605 9515074

[42] Bellini R, Carrieri M, Bacchi M, Fonti P, Celli G. Possible utilization of metallic copper to inhibit Aedes Albopictus (Skuse) larval development. J Am Mosq Control Assoc. 1998;14:451–6. 10084140

[43] Romi R, Di Luca M, Raineri W, Pesce M, Rey A, Giovannangeli S, Zanasi F, Bella A. Laboratory and field evaluation of metallic copper on Aedes albopictus (Diptera: Culicidae) larval development. J Med Entomol. 2000;37:281–5. doi: 10.1603/0022-2585-37.2.281 10730501

[44] Becker N, Oo TT, Schork N. Metallic copper spray–a new control technique to combat invasive container-inhabiting mosquitoes. Parasit Vectors. 2015;8:575. doi: 10.1186/s13071-015-1180-z 26553319 PMC4640347

[45] Reza M, Ilmiawati C. Laboratory testing of low concentration (<1 ppm) of copper to prolong mosquito pupation and adult emergence time: An alternative method to delay mosquito life cycle. PLoS One. 2020;15:e0226859. doi: 10.1371/journal.pone.0226859 32437348 PMC7241823

[46] Rodríguez MM, Bisset J, de Fernandez DM, Lauzán L, Soca A. Detection of insecticide resistance in Aedes aegypti (Diptera: Culicidae) from Cuba and Venezuela. J Med Entomol. 2001;38:623–8. doi: 10.1603/0022-2585-38.5.623 11580033

[47] Craig GBJ, Vandehey RC, Hickey WA. Genetic variability in populations of Aedes aegypti. Bull World Health Organ. 1961;24:527–39. 13696190 PMC2555912

[48] Tabachnick WJ. Geographic and temporal patterns of genetic variation of Aedes aegypti in New Orleans. Am J Trop Med Hyg. 1982;31:849–853. doi: 10.4269/ajtmh.1982.31.849 7102920

[49] Sim S, Jupatanakul N, Ramirez JL, Kang S, Romero-Vivas CM, Mohammed H, et al. Transcriptomic profiling of diverse Aedes aegypti strains reveals increased basal-level immune activation in dengue virus-refractory populations and identifies novel virus-vector molecular interactions. PLoS Negl Trop Dis. 2013;7:e2295. doi: 10.1371/journal.pntd.0002295 23861987 PMC3701703

[50] Zhu Y, Tong L, Nie K, Wiwatanaratanabutr I, Sun P, Li Q, et al. Host serum iron modulates dengue virus acquisition by mosquitoes. Nat Microbiol. 2019;4:2405–2415. doi: 10.1038/s41564-019-0555-x 31527795

[51] Licciardi S, Loire E, Cardinale E, Gislard M, Dubois E, Cêtre-Sossah C. In vitro shared transcriptomic responses of Aedes aegypti to arboviral infections: example of dengue and Rift Valley fever viruses. Parasit Vectors. 2020;13:395. doi: 10.1186/s13071-020-04253-5 32758286 PMC7404916

[52] Weber JJ, Kanost MR, Gorman MJ. Iron binding and release properties of transferrin-1 from Drosophila melanogaster and Manduca sexta: Implications for insect iron homeostasis. Insect Biochem Mol Biol. 2020;125:103438. doi: 10.1016/j.ibmb.2020.103438 32735914 PMC7501197

[53] Xiao G, Liu ZH, Zhao M, Wang HL, Zhou B. Transferrin 1 Functions in Iron Trafficking and Genetically Interacts with Ferritin in Drosophila melanogaster. Cell Rep. 2019;26:748–758.e5. doi: 10.1016/j.celrep.2018.12.053 30650364

[54] Iatsenko I, Marra A, Boquete JP, Peña J, Lemaitre B. Iron sequestration by transferrin 1 mediates nutritional immunity in Drosophila melanogaster. Proc Natl Acad Sci U S A. 2020;117:7317–7325. doi: 10.1073/pnas.1914830117 32188787 PMC7132258

[55] Zhou G, Velasquez LS, Geiser DL, Mayo JJ, Winzerling JJ. Differential regulation of transferrin 1 and 2 in Aedes aegypti. Insect Biochem Mol Biol. 2009;39:234–44. doi: 10.1016/j.ibmb.2008.12.004 19166934

[56] Chan AS, Rodríguez MH, Torres JA, Rodríguez Mdel C, Villarreal C. Susceptibility of three laboratory strains of Anopheles albimanus (Diptera: Culicidae) to coindigenous Plasmodium vivax in southern Mexico. J Med Entomol. 1994;31:400–3. doi: 10.1093/jmedent/31.3.400 8057314

[57] Georghiou GP, Gidden FE, Cameron JW. A stripe character in Anopheles albimanus (Diptera: Culicidae) and its linkage relationships to sex and dieldrin resistance. Ann Entomol Soc Am. 1967;60:323–8. doi: 10.1093/aesa/60.2.323 6044890

[58] Benedict MQ, Cohen A, Cornel AJ, Brummett DL. Uric Acid in Anopheles Mosquitoes (Diptera: Culicidae): Efects of Collarless, Stripe and White Mutations. Ann Entomol Soc Am. 1996;89:261–5.

[59] Claudio-Piedras F, Recio-Tótoro B, Condé R, Hernández-Tablas JM, Hurtado-Sil G, Lanz-Mendoza H. DNA Methylation in Anopheles albimanus Modulates the Midgut Immune Response Against Plasmodium berghei. Front Immunol. 2020;10:3025. doi: 10.3389/fimmu.2019.03025 31993053 PMC6970940

[60] Villarreal-Treviño C, Ríos-Delgado JC, Penilla-Navarro RP, Rodríguez AD, López JH, Nettel-Cruz JA, Moo-Llanes DA, Fuentes-Maldonado G. Composition and abundance of anopheline species according to habitat diversity in Mexico. Salud Publica Mex. 2020;62:388–401. doi: 10.21149/10111 32549083

[61] Weinberg ED, Moon J. Malaria and iron: history and review. Drug Metab Rev. 2009;41:644–62. doi: 10.1080/03602530903178905 19764831

[62] Spottiswoode N, Duffy PE, Drakesmith H. Iron, anemia and hepcidin in malaria. Front Pharmacol. 2014;5:125. doi: 10.3389/fphar.2014.00125 24910614 PMC4039013

[63] Raventos-Suarez C, Pollack S, Nagel RL. Plasmodium falciparum: inhibition of in vitro growth by desferrioxamine. Am J Trop Med Hyg. 1982;31:919–22. doi: 10.4269/ajtmh.1982.31.919 6751113

[64] Thipubon P, Uthaipibull C, Kamchonwongpaisan S, Tipsuwan W, Srichairatanakool S. Inhibitory effect of novel iron chelator, 1-(N-acetyl-6-aminohexyl)-3-hydroxy-2-methylpyridin-4-one (CM1) and green tea extract on growth of Plasmodium falciparum. Malar J. 2015;14:382. doi: 10.1186/s12936-015-0910-1 26424148 PMC4590262

[65] Fritsch G, Treumer J, Spira DT, Jung A. Plasmodium vinckei: suppression of mouse infections with desferrioxamine B. Exp Parasitol. 1985;60:171–4. doi: 10.1016/0014-4894(85)90020-7 4029347

[66] Ferrer P, Tripathi AK, Clark MA, Hand CC, Rienhoff HY Jr, Sullivan DJ Jr. Antimalarial iron chelator, FBS0701, shows asexual and gametocyte Plasmodium falciparum activity and single oral dose cure in a murine malaria model. PLoS One. 2012;7:e37171. doi: 10.1371/journal.pone.0037171 22629364 PMC3357340

[67] Pollack S, Rossan RN, Davidson DE, Escajadillo A. Desferrioxamine suppresses Plasmodium falciparum in Aotus monkeys. Proc Soc Exp Biol Med. 1987 Feb;184:162–4. doi: 10.3181/00379727-184-42461 3543939

[68] Gordeuk VR, Loyevsky M. Antimalarial effect of iron chelators. Adv Exp Med Biol. 2002;509:251–72. doi: 10.1007/978-1-4615-0593-8_13 12572998

[69] Smith HJ, Meremikwu M. Iron chelating agents for treating malaria. Cochrane Database Syst Rev. 2003;2:CD001474. doi: 10.1002/14651858.CD001474 10796645 PMC6532667

[70] Muriuki JM, Mentzer AJ, Mitchell R, Webb EL, Etyang AO, Kyobutungi C, et al. Malaria is a cause of iron deficiency in African children. Nat Med. 2021;27:653–658. doi: 10.1038/s41591-021-01238-4 33619371 PMC7610676

[71] Rasoloson D, Shi L, Chong CR, Kafsack BF, Sullivan DJ. Copper pathways in Plasmodium falciparum infected erythrocytes indicate an efflux role for the copper P-ATPase. Biochem J. 2004;381:803–11. doi: 10.1042/BJ20040335 15125686 PMC1133890

[72] Kenthirapalan S, Waters AP, Matuschewski K, Kooij TW. Copper-transporting ATPase is important for malaria parasite fertility. Mol Microbiol. 2014;91:315–25. doi: 10.1111/mmi.12461 24237419 PMC4016742

[73] Asahi H, Tolba ME, Tanabe M, Sugano S, Abe K, Kawamoto F. Perturbation of copper homeostasis is instrumental in early developmental arrest of intraerythrocytic Plasmodium falciparum. BMC Microbiol. 2014;14:167. doi: 10.1186/1471-2180-14-167 24961242 PMC4080775

[74] Gutiérrez L, Zubow K, Nield J, Gambis A, Mollereau B, Lázaro FJ, et al. Biophysical and genetic analysis of iron partitioning and ferritin function in Drosophila melanogaster. Metallomics. 2013;5:997–1005. doi: 10.1039/c3mt00118k 23771129

[75] Zhang B, Binks T, Burke R. The E3 ubiquitin ligase Slimb/beta-TrCP is required for normal copper homeostasis in Drosophila. Biochim Biophys Acta Mol Cell Res. 2020;1867: 118768. doi: 10.1016/j.bbamcr.2020.118768 32502619

[76] Ahmed AM, Hurd H. Immune stimulation and malaria infection impose reproductive costs in Anopheles gambiae via follicular apoptosis. Microbes Infect. 2006;8: 308–315. doi: 10.1016/j.micinf.2005.06.026 16213176

[77] Vézilier J, Nicot A, Gandon S, Rivero A. Plasmodium infection decreases fecundity and increases survival of mosquitoes. Proc Biol Sci. 2012;279:4033–4041. doi: 10.1098/rspb.2012.1394 22859589 PMC3427586

[78] Contreras-Garduño J, Rodríguez MC, Rodríguez MH, Alvarado-Delgado A, Lanz-Mendoza H. Cost of immune priming within generations: trade-off between infection and reproduction. Microbes Infect. 2014;16: 261–267. doi: 10.1016/j.micinf.2013.11.010 24291714

[79] Sadraie M, Missirlis F. Evidence for evolutionary constraints in Drosophila metal biology. Biometals. 2011;24:679–686. doi: 10.1007/s10534-011-9420-y 21293906

[80] Rempoulakis P, Afshar N, Osorio B, Barajas-Aceves M, Szular J, Ahmad S, et al. Conserved metallomics in two insect families evolving separately for a hundred million years. Biometals. 2014;27:1323–1335. doi: 10.1007/s10534-014-9793-9 25298233 PMC4223573

[81] Stobbart RH. Electrical potential differences and ionic transport in the larva of the mosquito Aedes aegypti (L.). J Exp Biol. 1974;60:493–533. 4832994 10.1242/jeb.60.2.493

[82] Patrick ML, Gonzalez RJ, Bradley TJ. Sodium and chloride regulation in freshwater and osmoconforming larvae of Culex mosquitoes. J Exp Biol. 2001;204:3345–3354. 11606608 10.1242/jeb.204.19.3345

[83] Dow JAT, Krause SA, Herzyk P. Updates on ion and water transport by the Malpighian tubule. Curr Opin Insect Sci. 2021; 47:31–37. doi: 10.1016/j.cois.2021.02.018 33705976 PMC9586879

[84] Blandin S, Shiao SH, Moita LF, Janse CJ, Waters AP, Kafatos FC, Levashina EA. Complement-like protein TEP1 is a determinant of vectorial capacity in the malaria vector Anopheles gambiae. Cell. 2004;116:661–670. doi: 10.1016/s0092-8674(04)00173-4 15006349

[85] Povelones M, Waterhouse RM, Kafatos FC, Christophides GK. Leucine-rich repeat protein complex activates mosquito complement in defense against Plasmodium parasites. Science. 2009;324:258–261. doi: 10.1126/science.1171400 19264986 PMC2790318

[86] Frolet C, Thoma M, Blandin S, Hoffmann JA, Levashina EA. Boosting NF-kappaB-dependent basal immunity of Anopheles gambiae aborts development of Plasmodium berghei. Immunity. 2006;25:677–685. doi: 10.1016/j.immuni.2006.08.019 17045818

[87] Vásquez-Procopio J, Rajpurohit S, Missirlis F. Cuticle darkening correlates with increased body copper content in Drosophila melanogaster. Biometals. 2020;33:293–303. doi: 10.1007/s10534-020-00245-1 33026606 PMC7538679

[88] Ward CM, Aumann RA, Whitehead MA, Nikolouli K, Leveque G, Gouvi G, et al.White pupae phenotype of tephritids is caused by parallel mutations of a MFS transporter. Nat Commun. 2021;12:491. doi: 10.1038/s41467-020-20680-5 33479218 PMC7820335

[89] Nan R, Tetchner S, Rodriguez E, Pao PJ, Gor J, Lengyel I, Perkins SJ. Zinc-induced self-association of complement C3b and Factor H: implications for inflammation and age-related macular degeneration. J Biol Chem. 2013;288:19197–19210. doi: 10.1074/jbc.M113.476143 23661701 PMC3696691

[90] Coverdale JPC, Barnett JP, Adamu AH, Griffiths EJ, Stewart AJ, Blindauer CA. A metalloproteomic analysis of interactions between plasma proteins and zinc: elevated fatty acid levels affect zinc distribution. Metallomics. 2019;11:1805–1819. doi: 10.1039/c9mt00177h 31612889

[91] Hernández-Martínez S, Cardoso-Jaime V, Nouzova M, Michalkova V, Ramirez CE, Fernandez-Lima F, et al. Juvenile hormone controls ovarian development in female Anopheles albimanus mosquitoes. Sci Reports. 2019; 9:2127. doi: 10.1038/s41598-019-38631-6 30765796 PMC6375968

[92] Franke-Fayard B, Trueman H, Ramesar J, Mendoza J, van der Keur M, van der Linden R, et al. A Plasmodium berghei reference line that constitutively expresses GFP at a high level throughout the complete life cycle. Mol Biochem Parasitol. 2004;137:23–33. doi: 10.1016/j.molbiopara.2004.04.007 15279948

[93] Recio-Tótoro B, Condé R, Claudio-Piedras F, Lanz-Mendoza H. Affinity purification of *Plasmodium* ookinetes from in vitro cultures using extracellular matrix gel. Parasitol Int. 2021;80:102242. doi: 10.1016/j.parint.2020.102242 33152548

