# Supplementary figures and images for: Mosquito metallomics reveal copper and iron as critical factors for Plasmodium infection
Source: PLoS Negl Trop Dis. 2021 Jun 23;15(6):e0009509. doi: 10.1371/journal.pntd.0009509 (PMC8221525; doi:10.1371/journal.pntd.0009509)

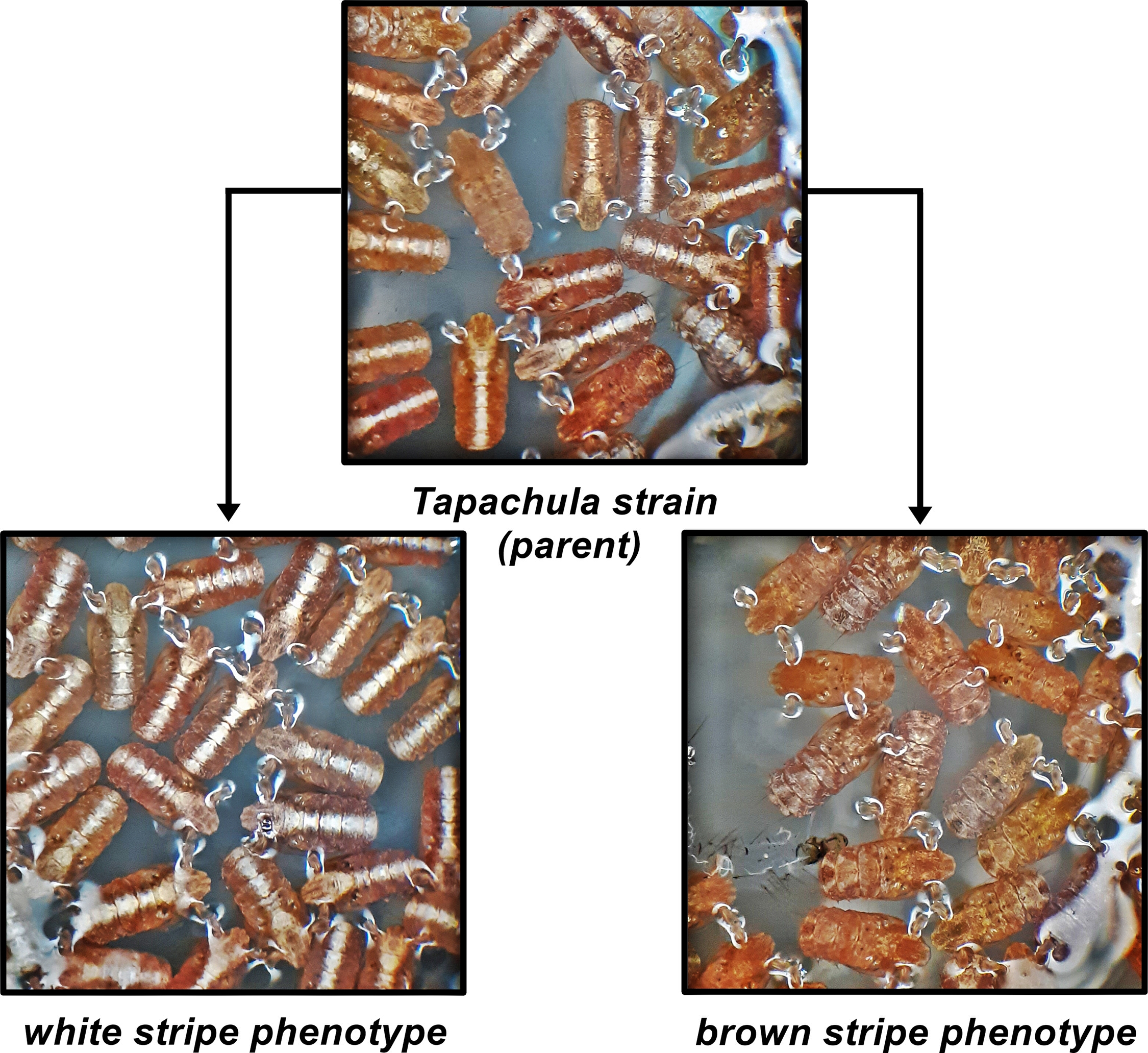

Supplement: S1 Fig — The parental strain contains both ws and bs phenotypes (top image). Following inbreeding for over sixty generations, the two phenotypes have been successfully separated (lower images). (TIF) [file pntd.0009509.s001.tif]

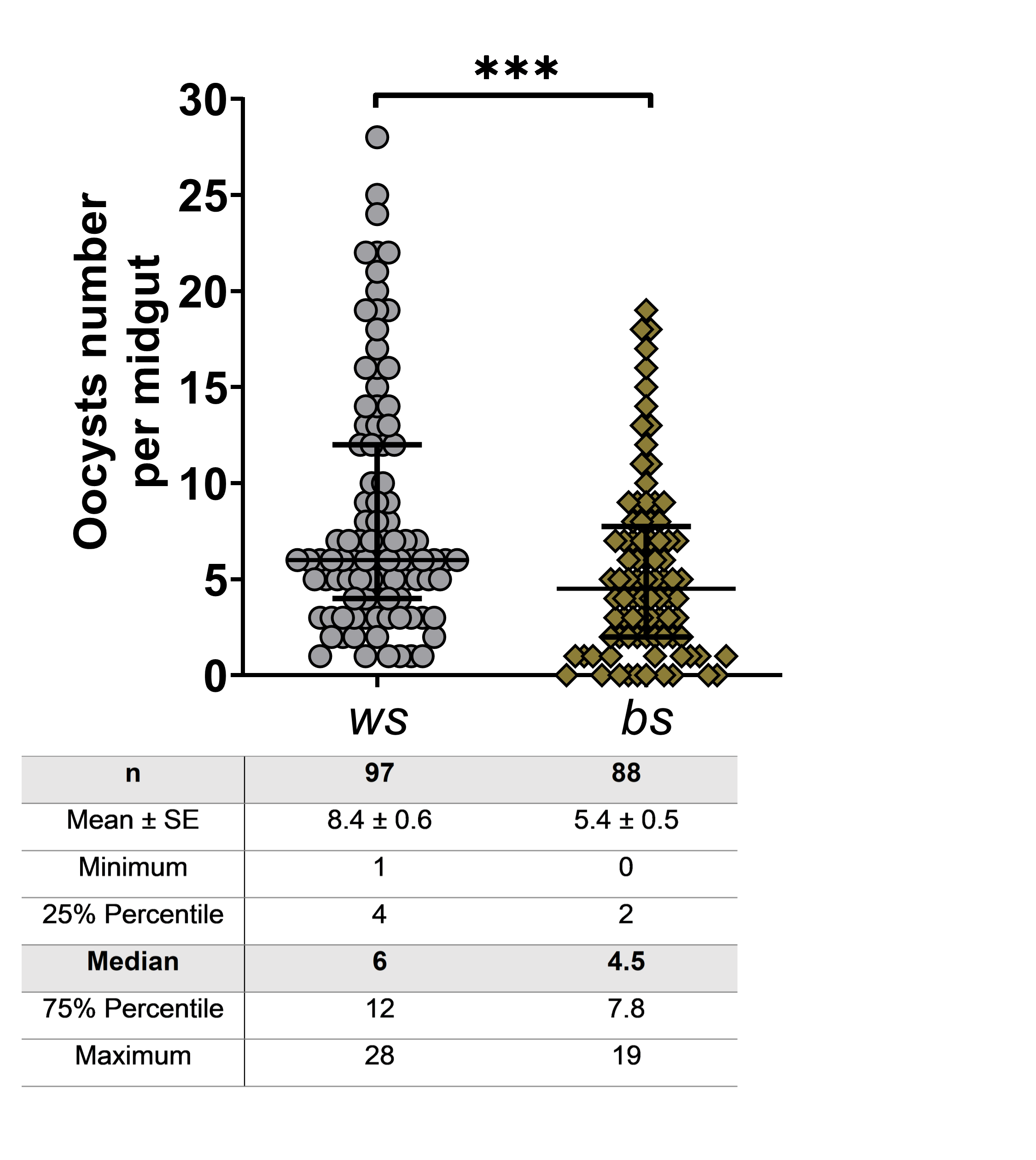

Supplement: S2 Fig — Oocyst number per midgut of ws (grey circles) and bs (brown diamonds) mosquitoes were determined at seven days post-feeding with P. berghei-infected blood. Data were analyzed with the Mann-Whitney U test, statistical difference is indicated by asterisks, ***p = 0.0006. Medians with interquartile range are indicated in black. Infection distributions for each phenotype are described in the table. (TIF) [file pntd.0009509.s002.tif]

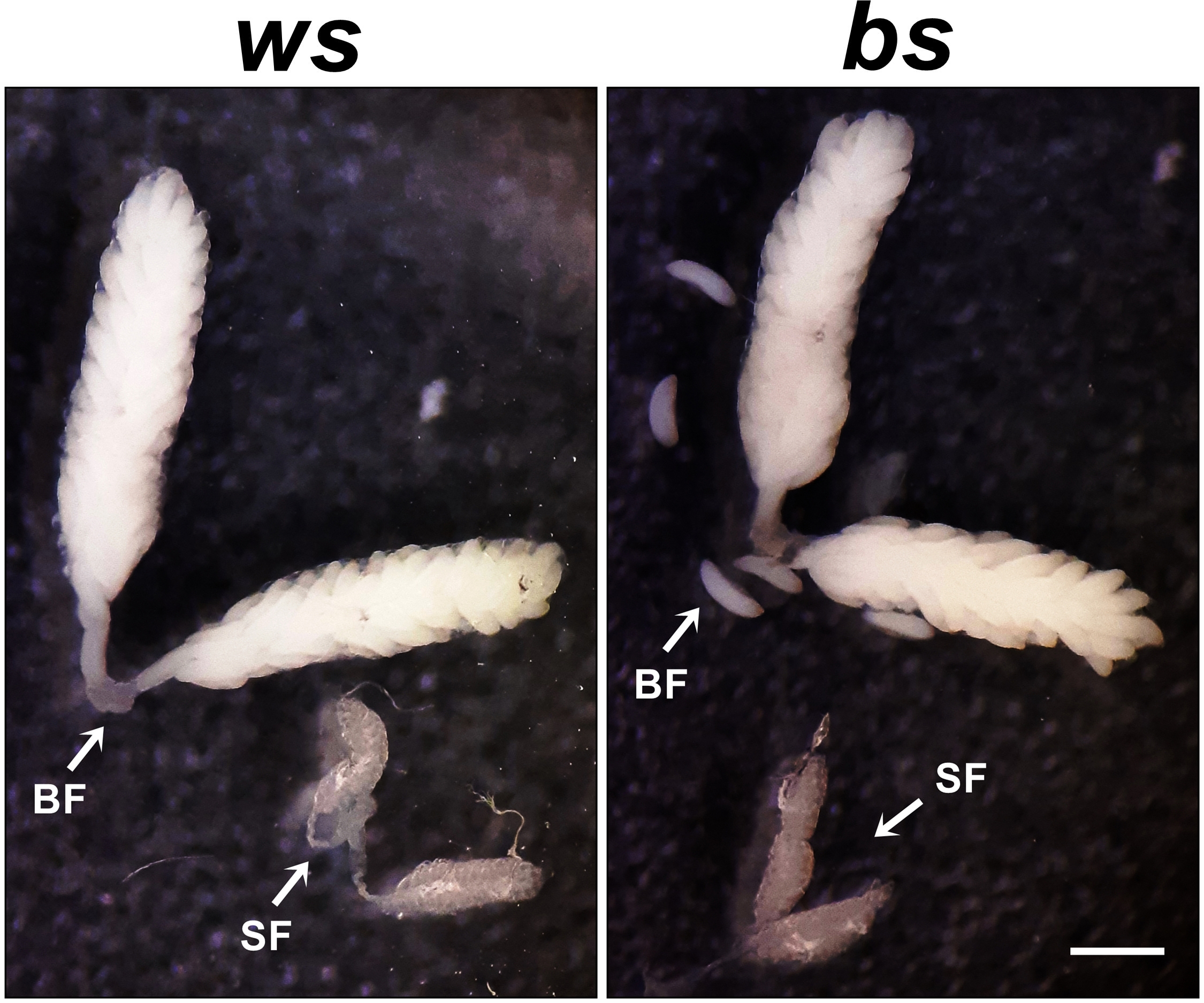

Supplement: S3 Fig — Dissected ovaries from ws (left) and bs (right) phenotypes of An. albimanus. After blood-feeding, ovarian development was observed in both phenotypes. Ovaries from blood-fed (BF) or sugar-fed (SF) mosquitoes are indicated with arrows. Scale bar = 500 μm. (TIF) [file pntd.0009509.s003.tif]
