# Supplementary material for: Mosquito metallomics reveal copper and iron as critical factors for Plasmodium infection
Source: PLoS Negl Trop Dis. 2021 Jun 23;15(6):e0009509. doi: 10.1371/journal.pntd.0009509 (PMC8221525; doi:10.1371/journal.pntd.0009509)
Supplement: S2 Table — Results are given in mg element per g of diet. The diet used for NO was also used for rearing of An. albimanus larvae. (DOCX) [file pntd.0009509.s005.docx]

**S2 Table. Elemental analysis of larval diets used in this study.**

Results are given in mg element per g of diet. The diet used for Rock was also used for rearing of *An. albimanus* larvae.

|  | **Fe mean ± SD** | **Cu mean ± SD** | **Zn mean ± SD** |
| --- | --- | --- | --- |
| **NO diet** | 0.21±0.03 | 0.02±0.01 | 0.12±0.01 |
| **Rock diet** | 0.24±0.07 | 0.02±0.00 | 0.11±0.00 |
| **p value (t-test)** | 0.48 | 0.46 | 0.24 |
|  | **Ca mean ± SD** | **Mg mean ± SD** | **Mn mean ± SD** |
| **NO diet** | 13.8±0.4 | 1.3±0.1 | 0.02±0.00 |
| **Rock diet** | 10.7±0.7 | 2.0±0.1 | 0.05±0.00 |
| **p value (t-test)** | <0.0001 | <0.0001 | <0.0001 |
|  | **Na mean ± SD** | **K mean ± SD** | **P mean ±SD** |
| **NO diet** | 4.6±0.3 | 7.7±0.2 | 7.9±0.5 |
| **Rock diet** | 4.2±0.2 | 6.4±0.1 | 4.9±0.3 |
| **p value (t-test)** | 0.05 | <0.0001 | <0.0001 |
