# Supplementary material for: Mosquito metallomics reveal copper and iron as critical factors for Plasmodium infection
Source: PLoS Negl Trop Dis. 2021 Jun 23;15(6):e0009509. doi: 10.1371/journal.pntd.0009509 (PMC8221525; doi:10.1371/journal.pntd.0009509)
Supplement: S4 Table — (DOCX) [file pntd.0009509.s007.docx]

**S4 Table. Infection distribution at seven days post *P. berghei* infection of *white* and *brown* phenotypes co-subjected to chelator treatments.**

|  | ***ws***  **control** | ***ws***  **+BPS** | ***ws***  **+BCS** | ***bs* control** | ***bs***  **+BPS** | ***bs***  **+BCS** |
| --- | --- | --- | --- | --- | --- | --- |
| **n** | 47 | 62 | 45 | 41 | 46 | 47 |
| **Mean ± SE** | **9.0 ±1.0** | **5.1 ±0.6** | **5.8 ±0.6** | **4.4 ±0.5** | **7.4 ±1.0** | **4.7 ±0.5** |
| Minimum | 0 | 0 | 0 | 0 | 0 | 0 |
| 25% Percentile | 4 | 2 | 2 | 1 | 3 | 2 |
| **Median** | **7** | **4** | **5** | **3** | **6** | **5** |
| 75% Percentile | 12 | 6 | 8.5 | 7 | 9 | 6 |
| Maximum | 36 | 27 | 17 | 13 | 29 | 13 |
